# The Metabolism and Growth of Web Forums

**DOI:** 10.1371/journal.pone.0102646

**Published:** 2014-08-12

**Authors:** Lingfei Wu, Jiang Zhang, Min Zhao

**Affiliations:** 1 Baidu Inc., Baidu Campus, Haidian District, Beijing, P. R. China; 2 School of Human Evolution and Social Change, Arizona State University, Tempe, Arizona, United States of America; 3 Center for the Study of Institutional Diversity, Arizona State University, Tempe, Arizona, United States of America; 4 School of Systems Science, Beijing Normal University, Beijing, P. R. China; Max Planck Institute for the Physics of Complex Systems, Germany

## Abstract

We view web forums as virtual living organisms feeding on user's clicks and investigate how they grow at the expense of clickstreams. We find that 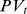 (the number of page views in a given time period) and 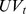 (the number of unique visitors in the time period) of the studied forums satisfy the law of the allometric growth, i.e., 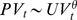. We construct clickstream networks and explain the observed temporal dynamics of networks by the interactions between nodes. We describe the transportation of clickstreams using the function 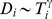, in which 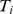 is the total amount of clickstreams passing through node 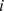 and 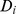 is the amount of the clickstreams dissipated from 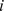 to the environment. It turns out that 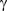, an indicator for the efficiency of network dissipation, not only negatively correlates with 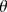, but also sets the bounds for 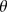. In particular, 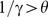 when 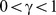 and 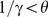 when 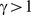. Our findings have practical consequences. For example, 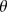 can be used as a measure of the “stickiness” of forums, which quantifies the stable ability of forums to remain users “lock-in” on the forum. Meanwhile, the correlation between 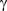 and 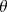 provides a method to predict the long-term “stickiness” of forums from the clickstream data in a short time period. Finally, we discuss a random walk model that replicates both of the allometric growth 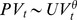 and the dissipation function 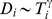.

## Introduction

A Web forum is an online discussion site allowing its members to exchange opinions by posting and replying threads. As one of the oldest Internet services, the user-generated-content nature of forums help them thrive in the era of Web 2.0 [1], [2]. The popularity of Web forums has motivated various studies on forum-based activities from detecting online opinion leaders [3] and analyzing political debates [4] to identifying interest-groups [5], [6]. Due to the challenge of collecting forum browsing data, previous studies usually focus on posting behavior and not browsing behavior. However, the forum usage analysis based on posting dynamics has strong limitations, because there are a large number of “silent” users who only read threads and do not give comments [7], [8].

In contrast with the lack of empirical studies on thread browsing, surfing behaviors in other online systems such as tagging sites [9] and social networking sites [7] have been extensively studied. A key concept in surfing dynamics is “clickstream”, which either refers to a series of webpages visited in a single session [10], or the successive clicks between two webpages generated by a group of users [11]. Most of early clickstream studies used this term at its first meaning and investigated the distribution of session length 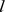 [7], [12] and its correlation with other variables, such as session duration [13] and user's log-off probability [14]. In particular, [15] proposed a novel interpretation of the mean value of 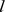 as “stickiness”, i.e., the ability of a site to keep visitors “lock-in”. With the development of network science, there is a trend to use “clickstream” at its second meaning in order to integrate clickstream studies and network theories into clickstream network analysis. In clickstream networks, nodes are information resources and edges are the successive clicks connecting resources [16]. As a general framework, clickstream network has been applied to model various online activities, such as photo tagging [9], news reading [17], and video watching [18]. As demonstrated by these studies, clickstream networks analysis provides novel interpretations to some well-studied problems [19]. For example, the surge and decay of news in the public domain is always understood as a result of the diffusion of information among users [20]. But from the perspective of clickstream networks, it can also be viewed as the transportation of user's attention between news [17].

In the current study we adopt the second definition of clickstream, that is, the successive clicks between two information resources, and use it as a quantification of collective attention online [11]. We get access to the historical data of Baidu Tieba, a very large Chinese Web Forum system, and systematically investigate the browsing activities of users on 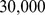 forums in two months. The size (average daily page views) of the studied forums varies from hundreds to millions. We also apply our analysis to two resource sharing forums, Delicous and Flickr, and compare them with Baidu Tieba. Different from previous studies that try to understand how users use forums, we propose to study how forums “consume” user's attention. Specifically, we view forums as “virtual living organisms” that grow at the expense of user's attention. In this perspective, we discuss the “metabolism” of forums, which describes how the attention of users are “absorbed” into and “dissipated” out from forums. Inspired by the metabolic theory of ecology [21]–[23], we compare the number of page views as the “body mass” of forums and the number of users as the “energy consumption”, and investigate how these two variables are related during the growth of forums. In data analysis, we track the anonymized “cookies”, which are permanent, unique identification labels of users, and count the number of unique cookies (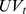) and page views (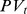) on an hourly basis. It turns out that the vast majority of the studied forums satisfy the allometric growth law 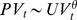, which means that the scaling exponent 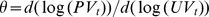 keeps unchanged over time. We suggest that 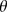 can be used to measure the “stickiness” of forums as an alternative to the average surfing length 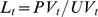 [15]. Because both of 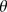 and 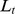 reflects the ability of forums to remain users “lock-in”, but the former is a constant over time, whereas the latter is not.

To probe into the origins of the allometric growth, we construct clickstream networks to define 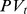 and 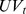 on these networks and explain the observed allometric growth by the interactions between nodes. In particular, we describe the dissipation of clickstreams on nodes using the scaling function 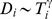 [24], [25]. And it turns out that 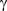, a quantity reflecting the network dissipation efficiency, is negatively correlated with 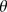. We also conduct a naive mathematical analysis to demonstrate how 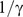 sets the upper and lower bounds for 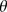. At the end of our study, we discuss a 2-D random walk model that replicates both of the scaling relationship between 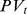 and 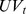 and the dissipation function connecting 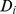 and 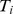.

Our study not only confirms the connection between growth and topology in complex systems [21], [26]–[28], but also has applied meanings. For example, the observed universal relationship between 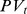 and 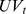 will help webmasters to benchmark and monitor the growth of different online communities. Meanwhile, the technique to predict the long-term behavior of forums by analyzing the random snapshots of clickstream networks may contribute to many areas of the Web development, such as click prediction [29] and interest group recommendation; 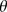 as a description of the “stickiness” of forums can be used as a novel feature in the recommendation of interest-groups [30]. Last but not least, we suggest that the presented clickstream network analysis actually provides a very general framework for studying user's browsing behavior in various online systems. To apply our analysis to other types of online social systems, one simply needs to replace the threads (nodes) with other information resources accordingly, such as news, tags, videos, etc.

## Materials and Methods

### Clickstream networks and key variables

**Figure 1:**
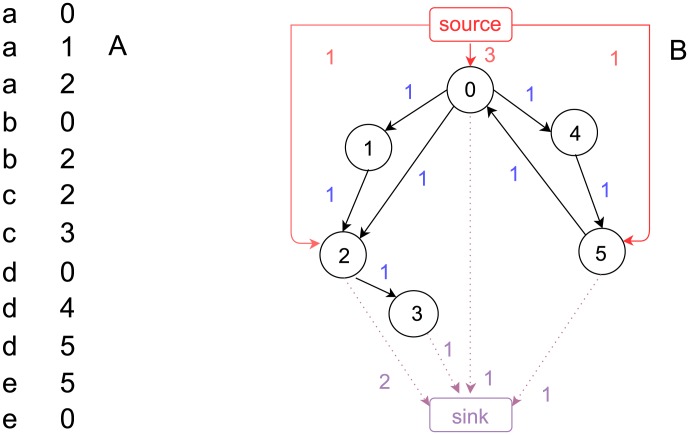
An example dataset of Baidu Tieba log file in one hour and the corresponding clickstream network. In (A) the left column shows the anonymized, sorted cookies and the right column shows the numeric ID of the visited threads. In (B) the nodes are threads and the weighted, directed links are user's switching between threads. The red arrows show the clickstreams “absorbed” from “source” and the purple, dotted arrows show the clickstreams “dissipated” to “sink”. In particular, the network in (B) is constructed as follows. For each record in the dataset, say, [a, 0], if the next record has the same cookie, e.g., [a, 1], we add a clickstream from node 0 to node 1; otherwise, we create a clickstream from node 0 to the artificially added node “sink”. After all records are converted into clickstreams, we add a “source” node to balance the network such that in-flow (weighted in-degree) equals out-flow (weighted out-degree) over all nodes except “source” and “sink” [43]. In the constructed networks, the values of passing-through clickstreams 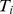 from node 0 to 5 are 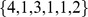, and the values of corresponding dissipation 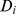 are 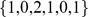. The values of 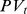 and 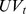 of this network are 12 and 5, respectively. Note that the value of 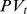 equals the total number of records in (A) and also the sum of 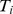, and the value of 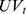 equals the total number of users in (A) and also the sum of 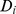.

Figure 1 presents an example Baidu Tieba clickstream network, whose nodes are threads and edges are user's switching between threads. The annotation of Figure 1 introduces how to construct clickstream networks from user's log files. We at first divide the entire data set into hourly pieces and then sort each piece by cookies (the unique and permanent labels used by a website to identify users). After that, we select all successive pairs of threads visited by the same user and connect them in the clickstream network. Sorting data by cookies guarantees that a user would not be repeatedly counted even if he is logged in/out more than once during a hour, so 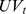 always represents the unique number of users.

We find that, after we adding “source” and “sink” to balance them [43], clickstream networks satisfy the constrain of “clickstream conservation”. Thus, 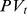 and 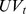 as network properties, can also be calculated at the node level. As network properties, 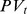 is the total weights of edges and 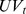 is the total clickstreams “dissipated” out of the network (i.e., the weighted in-degree of “sink”. Note that 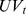 also equals the weighted out-degree of “source”, thus we can choose either “source” or “sink” to conduct the analysis. To make our clickstream networks comparable with ecological networks [24], we choose to analyze “sink”. See Figure S3 for the comparison between the dissipation behaviors calculated by “source” and “sink”). On the node level, 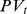 is the sum of the clickstreams passing through node 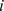 (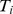) and 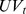 is the sum of the clickstreams dissipated by 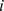 to “sink” (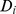):
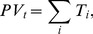
(1)




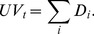
(2)


### Data

Two groups of data sets are used. The first one is the log file of Baidu Tieba (http://tieba.baidu.com/), a collection of many topic-specific forums. Among the millions of forums in the system, we select the top 30,000 forums, whose size (the averaged daily page views in two months) varies from hundreds to millions. For each forum, we construct 1,440 successive hourly-based clickstream networks using the historical browsing data in two months (from Feb. 27, 2013 to Apr. 27, 2013). The other group of data sets contains the historical log file of two popular tagging sites, Delicious (https://delicious.com) and Flickr (http://www.flickr.com). These two data sets are collected by the joint effort of the institutions in the TAGora European project (http://www.tagora-project.eu/data/), which have generated many papers including [9] and [38]. The Delicious data set covers individual tagging behavior in four years (from 2003-01-01 to 2006-12-28) and the Flickr data set covers tagging behavior in two years (from 2004-01-01 to 2005-12-31).

In constructing Flickr and Delicious clickstream networks, we use the same method as illustrated in Figure 1, except that the nodes (which were threads in Baidu networks) are now the tags used by users to annotate online resources and the links are the successive usage of two distinct tags. Meanwhile, although Tieba networks are constructed in an hourly basis, we construct Flickr and Delicious networks in a daily basis so that they all contain 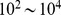 nodes and thus are comparable in size (see Figure S1). Despite these differences, our analysis shows that both types of clickstream networks exhibit very similar behaviors. Due to the data usage constraints, we are not able to release Tieba data. But we provide the download of Delicious and Flickr daily clickstream networks in http://pan.baidu.com/s/14Csma and http://pan.baidu.com/s/1gdsWMSN, respectively.

## Results

### The allometric growth of forums

Kleiber's law, or allometric growth, predicts that for a majority of living organisms, their energy consumption scales to body size with an exponent equals 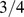 [21]. If we view online communities as virtual living organisms that feed on user's attention, a particularly interesting question would be, what are the counterparts of “body mass” and “energy consumption” of these virtual entities? Banavar et al. [27] explain Kleiber's law by modeling living organisms as flow networks that transport waters and nutrient. In their model, “body mass” is the total amount of flow circulating within a network and “energy consumption” is the amount of flow the network exchanges with the environment. By applying this model to clickstream networks, one would immediately find that these are also the definitions of “
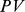
” (the total number of page views or clicks in a given period) and “
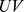
” (the total number of unique user sessions in the given period) of websites, respectively. Therefore, the online version of Kleiber's law, to exist, predicts that,
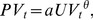
(3)in which 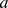 is a constant coefficient. The exponent 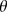 in Eq.3 not only shapes the growth dynamics of forums, but also provides a measure of the “stickiness” of forums as an alternative to the average surfing length 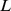, which is suggested in [15]. Using the indicator of 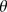, we can easily separate “sticky” forums from “non-sticky” forums. In particular, we derive that



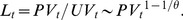
(4)If 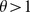 and hence 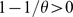, the average surfing length of users increases with forum size (or “body mass”). In other words, users are more likely to be “locked-in” in a forum during its growth. This is what we expect to see from a “sticky” forum. On the contrary, if 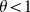 and hence 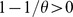, users on average navigate less threads as the size of the forum increases, which is the property of a “non-sticky” forum. An extra bonus of using 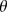 as the indicator is that, 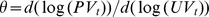 is a constant over time, whereas 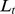 is obviously not. Therefore, 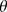 quantifies the “stickiness” of forums as a stable, long-term property.

**Figure 2:**
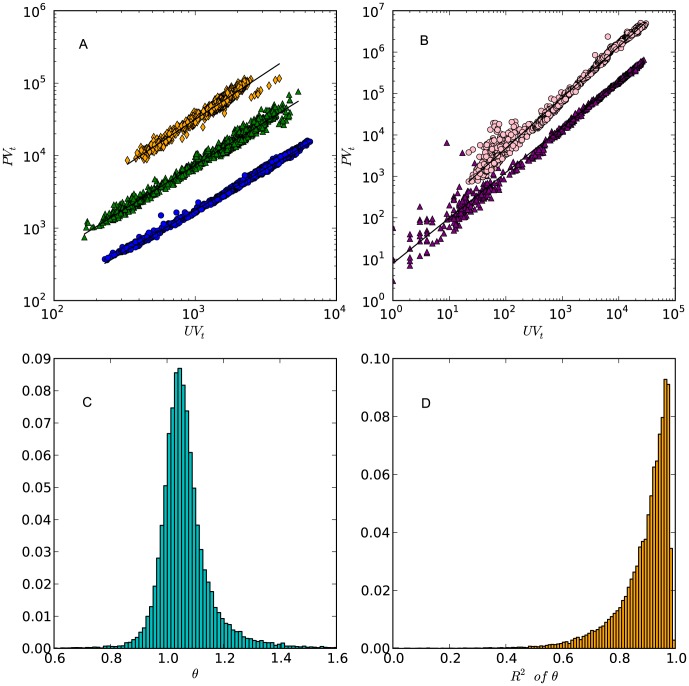
The scalings between 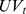 and 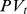 across three forums in 1,440 hours, (A). Each data point corresponds to a pair of 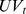 and 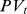 for an hourly network. Data points of different forums are shown in different colors. The values of 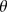 are 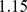 (blue circles), 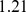 (green triangles), and 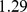 (orange diamonds), respectively. (B) The scalings between 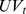 and 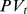 of Delicious (pink circles) and Flickr (purple triangles). Each data point corresponds to a pair of daily 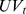 and 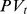. The values of 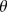 are 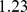 and 0.10, respectively. (C) The distribution of 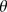 of 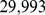 forums (the estimation of the rest 7 forums are removed due to a lack of data). The mean value is 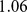 and the standard deviation (SD) is 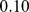. (D) The distribution of 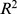 in fitting the 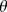 of Baidu forums. The mean value is 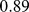 and the SD is 0.10.

Figure 2 demonstrates that Eq.3 characterizes the growth dynamics of three different forums and two tagging systems during the studied period. We find that this strong regularity holds for most of the studied forums: more than 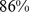 of forums have 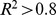 in the fitting of Eq.3. This finding suggests that the users of different forums obey similar behavioral logic in browsing threads collectively. It is very inspiring to find that human attention, after being quantified as clickstreams, satisfies the physical laws observed widely in natural flow systems [31].

In Kleiber's law, the “body mass” scales to “energy consumption” with an exponent 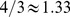 [21]. But the exponent observed in our data is generally smaller than this value. We conduct KS test [32] to verify the assumption that the calculated 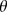 is drawn from a normal distribution with a mean equals 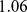 and a standard deviation (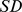) equals 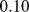. The p-value of the KS test is 0.07, suggesting that we can not reject this assumption. As shown by Figure 2C, the shape of the distribution is slightly asymmetrical; it skews towards the right hand side of the 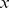 axis beyond the point of (x = 1, y = 0). In fact, 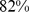 of the forums have a 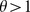. Thus most of the studied forums are “sticky”, in the sense that users are more likely to remain in the forums when the forums grow in size. However, by comparing 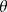 between virtual and real flow systems, we find that clickstream networks are still not as “sticky” as energy transportation networks within living organisms [21]. How can websites learn from living organisms? This is an interesting topic worth further exploration.

### The scaling of clickstream dissipation

We also discover an interesting scaling between 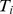 and 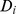 that describes the dissipation behavior of nodes,
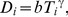
(5)in which 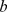 is a coefficient and 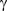 is an exponent that reflects the efficiency of network dissipation.

To understand the meaning of 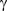, we can define the log-out probability of users on node 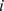 as
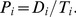
(6)


Thus, 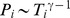 if Eq.5 holds. 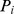 increases with the clickstreams passing-through nodes if 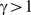, and decreases with the clickstreams otherwise. Therefore, the dissipation efficiency 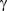 quantifies how the log-off probability changes with the node traffic 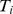.

**Figure 3:**
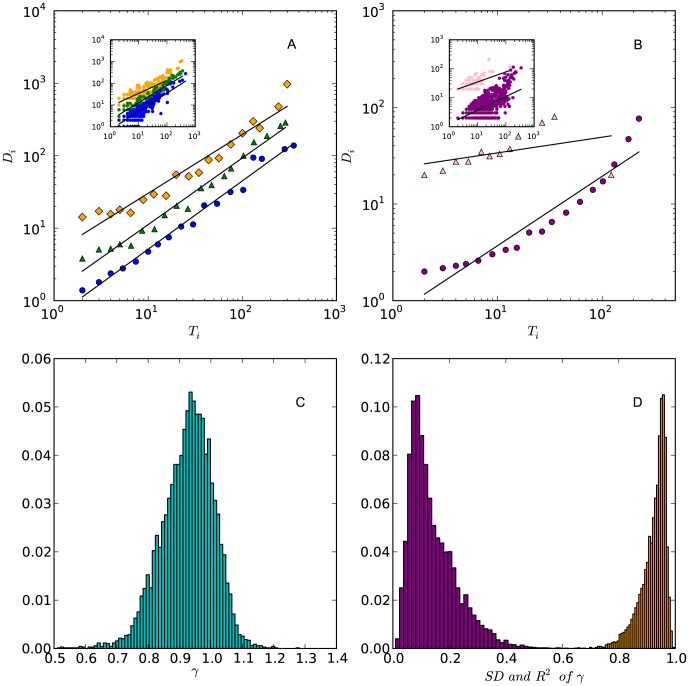
The scalings between 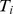 and 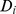 across forums in three hourly networks, (A). These three forums are the same as the forums presented in Figure 2A. The color scheme of these data points is the same as that of Figure 2A. The value of 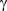 are 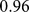 (blue circles), 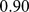 (green tringles), and 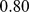 (orange diamonds) for the three forums, respectively. (B) The scalings between 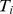 and 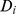 of Delicious (pink triangles) and Flickr (purple circles) in 2004-12-01. The values of 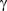 are 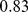 (Flickr) and 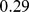 (Delicious), respectively. In both of (A) and (B), the regression estimation is applied on the log-binned data, in which we calculated the average of 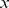 and 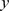 values in the intervals uniformly selected from the e-based logarithmic scaled 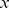 range. This technique is frequently used to eliminate the noise in data [44]. We also present the raw data in insets. (C) The distribution of the averaged value of 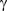 over 24 hours across 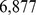 forums in Baidu Tieba. The mean value of the distribution is 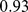 and the 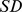 is 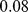. (D) The distribution of the 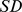 of 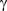 over 24 hours (purple bars) and the averaged 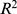 in fitting 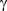 (orange bars). The mean and 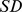 of the two distributions are 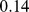, 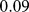, and 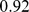,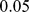, respectively.

Although 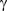 in Eq.5 seems to depend heavily on the flow structure of clickstreams networks, which may change in time, it is actually very stable during the growth of clickstream networks. We randomly select a day (Apr. 24, 2013) and construct 24 successive hourly networks for each of the studied 30,000 forums. We find that to estimate 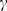, we just need one hourly clickstream network. The values of 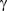 estimated from 24 networks have a very small standard deviation (SD). Figure 3 shows that more than 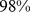 of forums have an 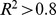 in the fitting of Eq.5. Meanwhile, the value of 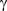 estimated from hourly networks is a stable quantity over time (the 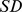 of 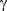
s in 24 hours is 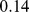). We conduct KS test to verify the assumption that the calculated mean value of 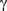 is drawn from a normal distribution with a mean equals 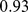 and an 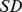 equals 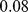. The p-value of the KS test is 0.14, suggesting that we can not reject this assumption. The distribution of 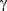 skews towards the left hand side of the 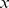 axis beyond the point of (x = 1, y = 0) and 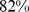 of forums have a value of 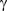 smaller than 1. According to aforementioned discussions, this means that most of the studied forums have a low dissipative efficiency, i.e., the log-out probability of users decreases with the clickstreams passing through threads.

This finding provides insight into the usage of Tieba forums by implying that users are more likely to log out from non-popular threads than popular threads. This is because Tieba system sorts threads in the reversed chronological order of comments and displays threads in multiple pages. Therefore, popular threads who receive more comments always appear on the first page. Unlike News aggregators such as Yahoo!, Tieba is an interested-based community containing topic-specific forums, therefore instead of selective reading, users usually simply browse the threads one by one in the default displaying order. As a result, when users get tired, they usually have read the most popular threads.

### The negative correlation between 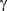 and 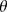


**Figure 4:**
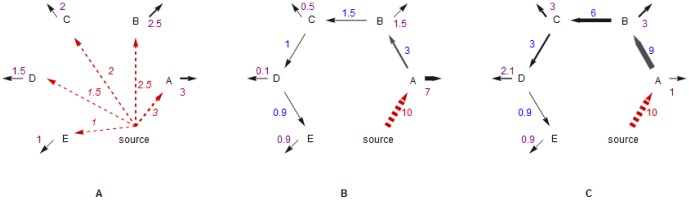
Three example clickstream networks of different topologies. The dashed red arrows show the clickstreams from source to the other nodes in the network. The black arrows show the transportation of clickstreams within the networks (the weights of which are shown in blue letters) and the dissipation of the clickstreams out of the networks (the weights of which are shown in purple letters). (A) A star-like network in which the dissipation probability of all nodes equals 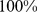. (B) A chain-like network in which the dissipation probability 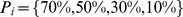 decreases from node A to D. As the clickstreams passing though nodes (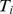) also decreases from A to D, 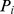 is positively correlated with 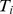. (C) A chain-like network in which the dissipation probability 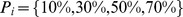 increases from node A to D. 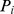 is negatively correlated with 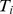. According to Eq.5 and Eq.6, we are able to derive that 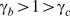. As we also know that 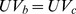 and 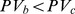, which implies that 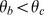, we find that 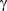 and 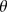 are negatively correlated. In the above naive comparison, we ignore the behavior of node E, whose traffic is very small compared to the other nodes.

By reviewing Eq.3 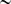 Eq.5, one would naturally expect that the dissipation efficiency 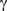 and the stickiness 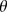 are related. To understand the connection between the two parameters, let's consider two extreme topologies, the star-like (Figure 4A) and the chain-like (Figure 4B 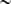
C). In the star-like topology, threads (nodes) receive clickstreams directly from the “environment” and dissipate them immediately, whereas in the chain-like topology, threads transport clickstreams from one to another and dissipate a portion of clickstreams in each step. If we fix the 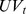 of the three clickstream networks to be the same as 10 units, we will find that the resulting 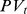 is different: it is larger in the chain-like networks (10+3+1.5+1+0.9 = 16.4 in B and 10+9+6+3+0.9 = 28.9 in C) than in the star-like network (3+2.5+1.5+1 = 10 in A). This is because by transporting clickstreams a network increases its storage capacity of clickstreams, i.e., the “body mass”.

To understand this interesting phenomenon, one can consider how a clown plays balls. A clown can barely hold more than two balls if he just grasps them in his hands, but he can easily maintain a circulation of many balls by throwing them up and passing them between hands. It is in exactly the same way that clickstream transportation increases the total amount of clickstreams “hold” by a network.

Furthermore, we find that a small 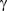 would decrease the dissipation of clickstreams and thus increases the network storage capacity. This finding is demonstrated by the comparison between Figure 4B and C. We calculate that 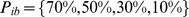 from node 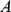 to 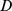 in Figure 4B and 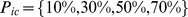 in Figure 4C (for the convenience of the comparison, we ignore the behavior of node E, whose traffic is very small compared to other nodes). As the pass-through clickstreams decrease monotonously from 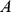 to 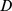, it is easy to derive that 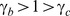. Recalling the conclusions that 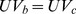 and 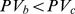, which imply that 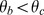, we find that 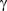 and 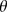 are negatively correlated. In fact, it is reasonable to expect this negative correlation being applicable to clickstream networks of all kinds of topologies. Because a small 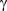 will always force large nodes to transport clickstreams to other nodes rather than dissipating them to the environment.

**Figure 5:**
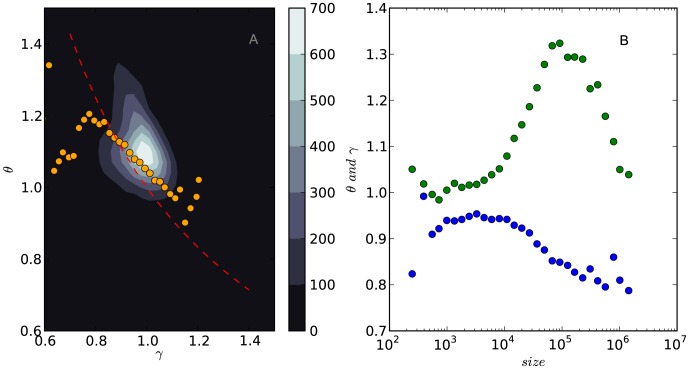
The negative correlation between 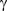 and 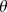 (A) and the change of 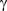 (blue circles) and 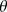 (green circles) with forum size (B). In (A) We plot both of the linear-binned data (orange circles) and the original data (heat map) and in (B) we only show the linear-binned data. In the heat map, the lighter color means that the distribution of the data points is more dense. The ticks on the color bar show the number of data points within a 0.1*0.1 square.

Figure 5A shows that the empirical data support the negative correlation between 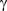 and 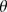. To summarize, the reversed chronological displaying order of threads seems to decrease the dissipation efficiency 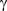 and increase the “stickiness” 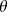 of the studied forums. This may be the reason why such displaying order is so common among forums. The web masters may or may not have noticed that, this strategy beats its competitors by generating a flow structure that attracts more users and thus spreads out in the evolution of forums.

As a complementary analysis, we also examine whether 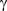 and 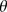 are affected by the forum size. We plot these two quantities against forum size in Figure 5B and find that when the forum size approximates 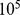 daily views, 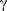 reaches its minimum value and 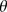 reaches its maximum value. This observation can be used to benchmark the growth of Tieba forums.

### 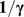 as the Bound of 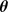


Negative correlation is not the only connection between 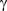 and 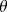. Here we present some derivations to demonstrate that 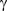 actually sets the bounds for 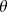. We can put Eq. 3, Eq. 1, Eq. 2, and Eq. 5 together as
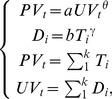
(7)in which 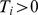 and 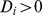.

If 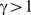, then 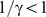. Assuming that there are 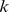 nodes in the network, we can derive that (see SI for the derivation in details)
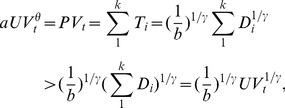
(8)and that



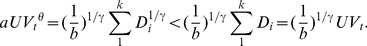
(9)Putting Eq. 8 and Eq. 9 together we have
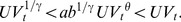
(10)


Compare to 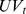, whose value varies from 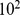 to 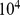 (Figure 2), the value of 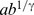, which varies from 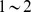 (see Figure S2), is very small. Therefore, the following inequality should be satisfied to guarantee Eq. 10:
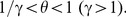
(11)


Similarly, when 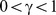 and thus 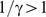 we can derive that
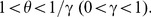
(12)


We find that Eq. 11 and Eq. 12 are supported by Figure 5. When 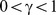, 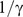 (the red, dotted line) is the upper bound of the expect values of 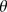 (the orange circles); when 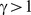, 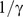 becomes the lower bound.

## Discussion and Conclusion

### A model of individual surfing behavior

We propose a simple model that replicates the observed two scaling laws (Eq. 3 and Eq. 5). Two properties of surfing behavior feature our model: 1) users can both browse existed threads and also publish new threads; 2) the previous surfing activities have an effect on the following surfing activities.

**Figure 6:**
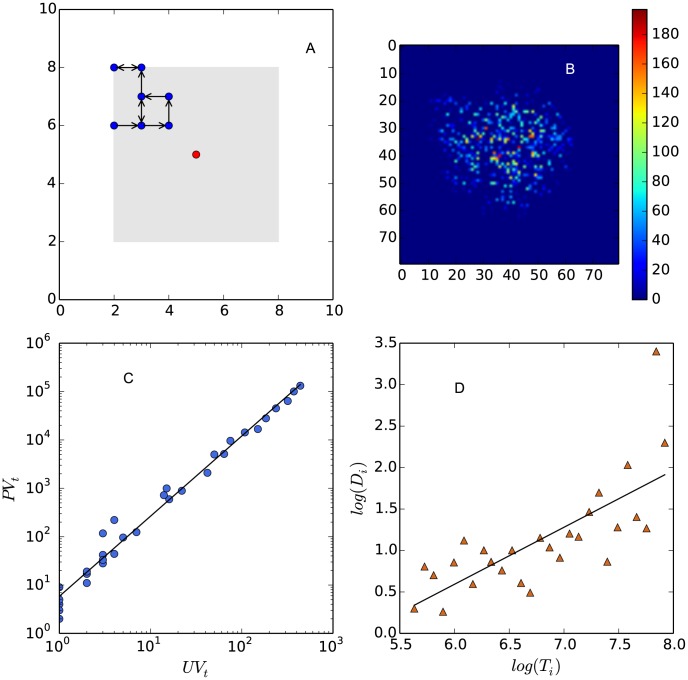
A model of individual surfing behavior. (A) The arrows show the trajectory of a random walker who starts from (2, 6) and ends in (3, 8). New threads (blue points) are created with probability 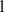 by the random walks. The walker stops when he goes beyond the “observation zone” (the gray square) of the existed threads generated in the last iteration of simulation. To initialize the simulation, we place a seed thread in the center of the grid as the existed thread (the red point). (B) The 1,000 generated threads (85 iterations, 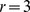, 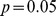) within a 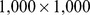 space. At the beginning of the simulation, there is only one thread at the center of the space. For each iteration, we throw 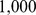 particles uniformly distributed into the space. We use warmer colors to show the larger number of visits to threads. In this plot we only show the central part of the original space in order to obtain a higher resolution network structure. (C) The increase of the total number of repeatedly visited threads (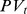) with the number of user sessions (random walks) (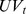). Each data point corresponds to an iteration in the simulation. The scaling exponent 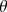 is 1.63. (D) The increase of the number of particles leaving the system from thread 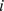 (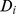) with the number of total visits to 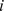 (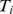), both axes are shown in e-based logarithmic scale. The exponent 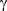 is 0.77.

Specifically, we model surfing activities by random walks within a 2-D grid containing randomly distributed threads. The Euclidean distances between threads indicate their similarities. To initialize the simulation we place only one thread (the red point in Figure 6 A) as a seed at the center of the universe. In each iteration we drop an fixed number of users uniformly to the system, who will walk randomly on the grid and create new threads with probability 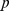 until there are no existed threads within their “observation zones” (an 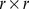 square around their current position). This is to represent that users will leave the forum if they can not find interested threads within a search area. We place no constraint on user's random walk so a thread may be visited repeatedly. But if a user can not find existed threads within the “observation zone” at the first step of his random walk, he will leave the system immediately and does not contribute to the statistics mentioned in the following part.

A random walk in this model represents the browsing activities generated by a user in a given time period. Therefore, the number of random walks can be expressed as 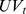 and the total number of repeatedly visited threads is 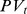. As time goes by, threads are created and are connected by user's random walks, leading to a growing clickstream network on the grid (Figure 6 B), which attracts more users and allows longer random walks. If we define 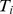 as the total number of random walks visiting thread 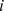 and 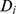 as the number of particles leaving the system from 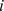, we will find that Eq. 1 and Eq. 2 still hold. This is because there is also “clickstream conservation” in this model; the number of users entering into the system equals the sum of users leaving the system over all nodes, and the total number of repeatedly visited threads equals the sum of visits to each thread. As shown by Figure 6 C and D, our model demonstrated both of the allometric growth (Eq. 3) and the scaling law of dissipation (Eq. 5). Our model also exhibits the negative correlation between 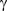 and 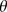 (see Figure S4), although this relationship is not significant (Pearson correlation coefficient equals −0.23 and p-value equals 0.5).

We conjecture that, the observed super-linear scaling between 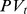 and 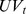 in our model originates from the fractal flow network structures [26] at the early stage of the simulation. A strong limitation of our model is that, as time passes, this fractal structure converges to a completely filled 2-D disk. This explains why the scaling exponent 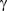 evolves towards 2 (in theory, a random walker can visit any point within a 2-D space, so the average length 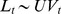 and hence 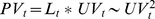) and also why the dissipation exponent 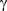 evolves towards 0 (eventually the dissipation only happens on the edge of the disk, so the average dissipation of all threads on disk approaches 0).

The novel feature of this model is that it demonstrates how flow creates a structure that attracts more flow. The co-evolution between structure and flow makes this model very different from previous network models, which either focus on the dynamics of networks [33], [34] or the dynamics on networks [20], but not both of them.

### The distribution of forum categories in the 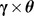 space

**Figure 7:**
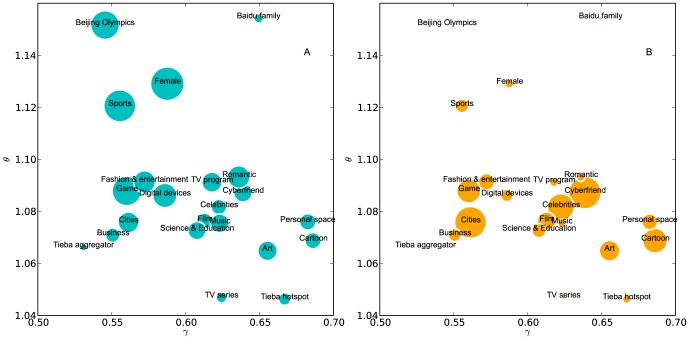
The mean values of 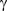 and 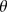 of different categories of forums of Baidu Tieba. Each circle corresponds to a category of forum. The size of green circles in (A) reflects the average size of forums in the category and the size of orange circles in (B) shows the number of forums within the category.

Driven by practical interests, we investigate whether the content of forums relates to their stickiness and dissipation efficiency. Figure 7 gives the distribution of 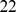 categories of 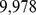 forums (the rest of the top 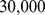 forums are removed due to a lack of human labeling data) in the 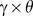 space. Each circle corresponds to a category of forums labeled by human coders. The size of green and orange circles reflects the average size and the number of forums in the corresponding category, respectively. We observe that 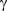 and 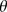 are negatively correlated, which is consistent with the findings in Figure 5.

This analysis provides insights for the management of Tieba forums. For example, the categories “Art”, “Cartoon”, and “Personal space” locate at the lower-right corner of the space, suffering from high dissipation efficiency and low stickiness. It means that on these forums users do not read a lot of threads within a single session. On the contrary, the categories “Beijing Olympics”, “female”, and “sports” have high stickiness, suggesting that users to these forums are generating more clicks. In particular, the high value of 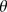 of the “Female” category suggests that there are a lot of female Tieba users. This conclusion is supported by the user statistics of Alexa (www.alexa.com), which suggests that the proportion of female users of Baidu Tieba is higher than the average level of the Web users.

### Summary

Websites, by their very nature, are the consumers of collective attention and the producers of information [35]. The comparison of websites as living organisms is not just a qualitative metaphor, but also provides quantitative insights into the understanding of websites development. In this study, we find substantial evidence that the growth dynamics of websites is governed by laws that are known to shape the evolution of natural flow systems [21].

In particular, we discuss the online version of Kleibers' law, that is, the scaling between 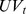 and 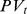 in the temporal evolution of forums. Furthermore, we show that the allometric exponent 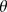, which is an indicator for the “stickiness” of forums in attracting users, is determined by the metabolism of clickstream networks. The lower the dissipation efficiency 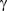 is, the larger the 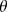 would be. Interestingly, there seems to be an optimized scale of forums at around 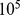 daily 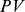
s that minimizes 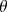 and maximizes 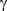. Finally, we discussed a random walk model that replicates both of the allometric growth and the dissipation patterns.

As suggested by Bettencourt et al. [36], the allometric growth is a very general relationship between variables in the evolution of complex systems. In particular, they show that cities are extensions of biological entities, in the sense that they satisfy the same allometric functions [22], [36]. Our study extends their findings from offline social systems to online social systems. We are not the only researchers who have noticed the scaling laws in online communities. For example, the recently found “densification” pattern in the growth of online networks [37], together with the scalings discussed in [37]–[41], are different versions of the “allometric growth” of online flow networks.

Our findings are relevant to the Web development in many aspects. In particular, the presented method predicts the long-term trend of clicks thus is useful in computational advertisement [42]. To predict the “stickiness” 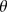 of forums, one just need to collect a random sample of threads and record the clickstreams passing through and being dissipated by them in a single hour. Another possible application of 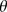 is to use it as a novel feature in the recommendation of interest-based groups [30].

## Supporting Information

Figure S1 **Three snapshots of clickstream networks of Delicious.** (A), (B), and (C) show the networks in 2003-01-01, 2003-06-01, and 2003-12-01, respectively. In each network, the nodes are tags and the weighted links are the sequential usage of two tags by users. In these networks source and sink are denoted by green and red colors, respectively. Other nodes are clustered by the week components they belong to and the nodes from the same cluster are shown in the same color. The networks are connected by source and sink and will fall apart if we remove these two nodes. It is observed that as the networks evolve, the largest component (in blue color) grows, connecting frequently used tags.
(EPS)

Figure S2 **The empirical distributions of the parameters of Eq. 8.** The value of *a* of each forum is estimated from the scaling relationship between *UV_t_* and *PV_t_* (see Eq. 8) in 1440 hours. In estimating the values of *b* we construct 1440 hourly flow networks for each forum, estimate the hourly scaling exponent between *T_i_* and *D_i_* (also see Eq. 8), and calculate the mean of the hourly values. The distributions shows the parameters for the top 1,000 forums.
(EPS)

Figure S3
**The linear relationship between** 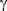 **_D_ and** 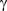 **_I_.** We plot both of the \binned” data (orange circles) and the original data (heat map). In the heat map, the lighter color means that the distribution of the data points is more dense. The slope of the regression line fitted from the binned data is 0∶46.(EPS)

Figure S4
**Some results of simulation.** (A) The change of 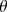 (blue points) and 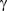 (red points) with the thread generating probability *p*. (B) The negative correlation between 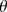 and 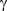. The Pearson correlation between the 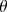 and 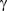 is −0.23, which is consistent with the empirical findings. However, this estimation has a p-value equals 0.5, thus we fail to significantly rule out the probability that the two parameters are independent. Simulations on the larger scales are needed to conform the relationship between 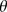 and 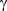 in this model.(EPS)
